# Site-Dependent Degradation of a Non-Cleavable Auristatin-Based Linker-Payload in Rodent Plasma and Its Effect on ADC Efficacy

**DOI:** 10.1371/journal.pone.0132282

**Published:** 2015-07-10

**Authors:** Magdalena Dorywalska, Pavel Strop, Jody A. Melton-Witt, Adela Hasa-Moreno, Santiago E. Farias, Meritxell Galindo Casas, Kathy Delaria, Victor Lui, Kris Poulsen, Janette Sutton, Gary Bolton, Dahui Zhou, Ludivine Moine, Russell Dushin, Thomas-Toan Tran, Shu-Hui Liu, Mathias Rickert, Davide Foletti, David L. Shelton, Jaume Pons, Arvind Rajpal

**Affiliations:** 1 Rinat Laboratories, Pfizer Inc., 230 East Grand Avenue, South San Francisco, CA, 94080, United States of America; 2 Worldwide Medicinal Chemistry, Pfizer Inc., 445 Eastern Point Road, Groton, CT, 06340, United States of America; Weizmann Institute of Science, ISRAEL

## Abstract

The efficacy of an antibody-drug conjugate (ADC) is dependent on the properties of its linker-payload which must remain stable while in systemic circulation but undergo efficient processing upon internalization into target cells. Here, we examine the stability of a non-cleavable Amino-PEG6-based linker bearing the monomethyl auristatin D (MMAD) payload site-specifically conjugated at multiple positions on an antibody. Enzymatic conjugation with transglutaminase allows us to create a stable amide linkage that remains intact across all tested conjugation sites on the antibody, and provides us with an opportunity to examine the stability of the auristatin payload itself. We report a position-dependent degradation of the C terminus of MMAD in rodent plasma that has a detrimental effect on its potency. The MMAD cleavage can be eliminated by either modifying the C terminus of the toxin, or by selection of conjugation site. Both approaches result in improved stability and potency *in vitro* and *in vivo*. Furthermore, we show that the MMAD metabolism in mouse plasma is likely mediated by a serine-based hydrolase, appears much less pronounced in rat, and was not detected in cynomolgus monkey or human plasma. Clarifying these species differences and controlling toxin degradation to optimize ADC stability in rodents is essential to make the best ADC selection from preclinical models. The data presented here demonstrate that site selection and toxin susceptibility to mouse plasma degradation are important considerations in the design of non-cleavable ADCs, and further highlight the benefits of site-specific conjugation methods.

## Introduction

Antibody-drug conjugates are a promising class of antibody-based therapeutics that take advantage of antibody specificity and small molecule cytotoxicity. The combined approach can deliver cytotoxic agents more specifically to the target cells and minimize harmful side effects in anti-tumor treatments [1–3]. The cytotoxic drugs are typically linked to the antibody carrier through either cleavable or non-cleavable linkers. Cleavable linkers contain a trigger that can release the cytotoxic agent after internalization and can utilize a change in pH, redox potential, or a proteolytic or other enzymatic event for linker processing [4, 5]. Non-cleavable linkers, on the other hand, rely on the degradation of the antibody itself to release the drug-linker, typically with the amino acid residue it was conjugated to as the released species [4, 6]. The choice of linker is one of the critical parameters of ADC design, and is usually experimentally determined for each target and cell type. Numerous examples of both linker types (cleavable and non-cleavable) are currently utilized in the clinic [7–9]. Regardless of its type, the ideal linker remains intact in systemic circulation, and releases the cytotoxic agent only after internalization in the target cell [9, 10], either by the built-in trigger (cleavable linkers) or by degradation of the antibody (non-cleavable linkers).

A number of non-cleavable linkers have been developed based on traditional conjugation methods using maleimide or N-hydroxysuccinimide ester coupling chemistry. The maleimide chemistry, although more specific due to lower number of potential target sites on the antibody, has been reported to uncouple in circulation, resulting in decreased exposure of the ADC relative to the total antibody [11–13]. Recently, several ways of reducing maleimide-based decoupling have been reported [12, 14, 15], overcoming a hurdle to future clinical development of those linkers.

An alternative site-specific conjugation method using microbial transglutaminase permits the direct introduction of fully assembled linker-payloads and fluorescent probes at a wide range of positions on the antibody [16–20]. In this approach, the enzymatic transamidation reaction creates a covalent linkage between the γ-carboxamide group of a native or engineered glutamine on the antibody and the primary amine on the linker-payload [16–18]. Previously, we investigated the dependence of cleavable linker stability on the site of conjugation, and identified distinct sites conferring most protection from cleavage in rodent plasma [17, 21]. In this report, we focus on the systematic characterization of transglutaminase-generated ADCs bearing non-cleavable auristatin-based linker-payloads at previously identified sites. We show that the glutamine tags engineered into the antibody sequence, as well as the transglutaminase-catalyzed isopeptide linkage between the engineered antibody and the payload remain intact upon exposure to mouse, rat, cynomolgus monkey, and human plasma *in vitro*, and upon administration in mouse *in vivo*. While the transglutaminase linkage to the antibody remains intact, we detect degradation of the C terminus of MMAD in rodent plasma. We investigate the dependence of MMAD stability on the site of conjugation, and correlate the stability with efficacy *in vitro* and *in vivo*. We further examine the observed degradation of MMAD to gain a mechanistic insight into this process.

## Results and Discussion

**Fig 1:**
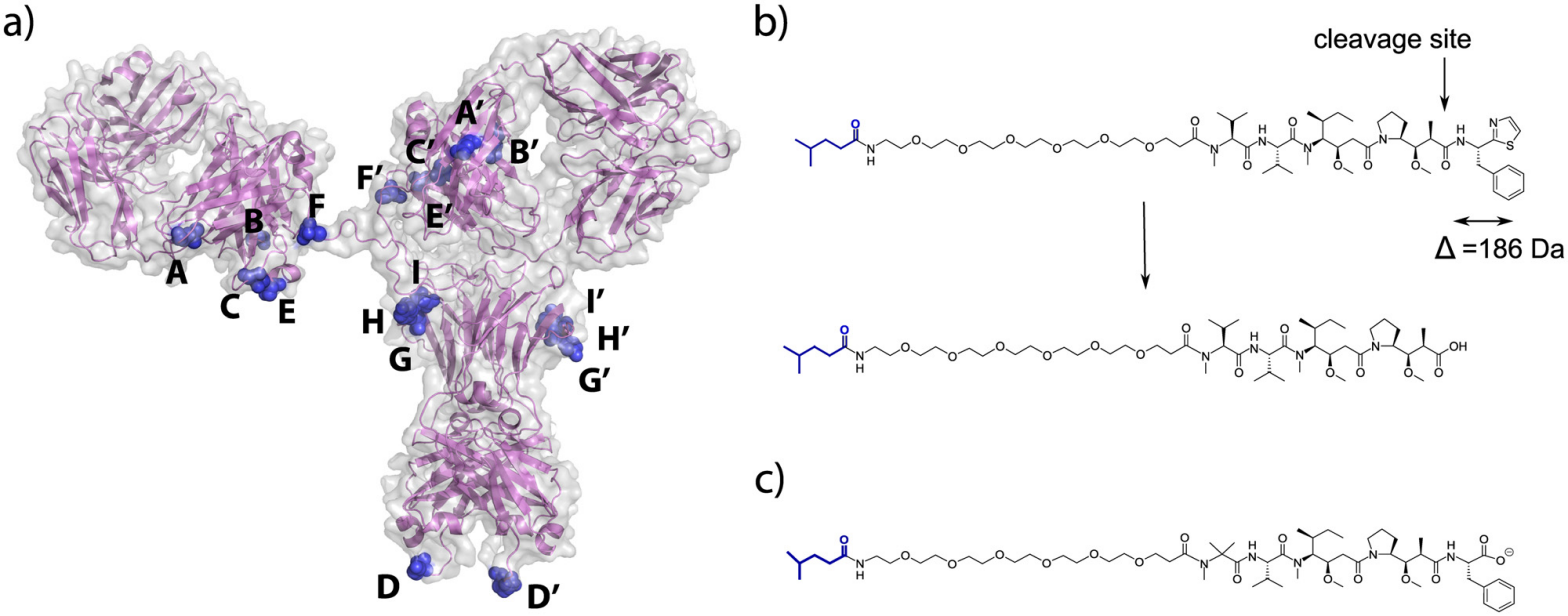
Stability studies of site-specific non-cleavable ADCs. a) Positions of conjugation sites on an antibody. b) Structure of the PEG6-C2-MMAD non-cleavable payload conjugated to the glutamine tag on the antibody, and its cleavage product. The glutamine residue is shown in blue. c) Structure of the PEG6-C2-Aur3377 non-cleavable payload conjugated to the glutamine tag shown in blue.

**Fig 2:**
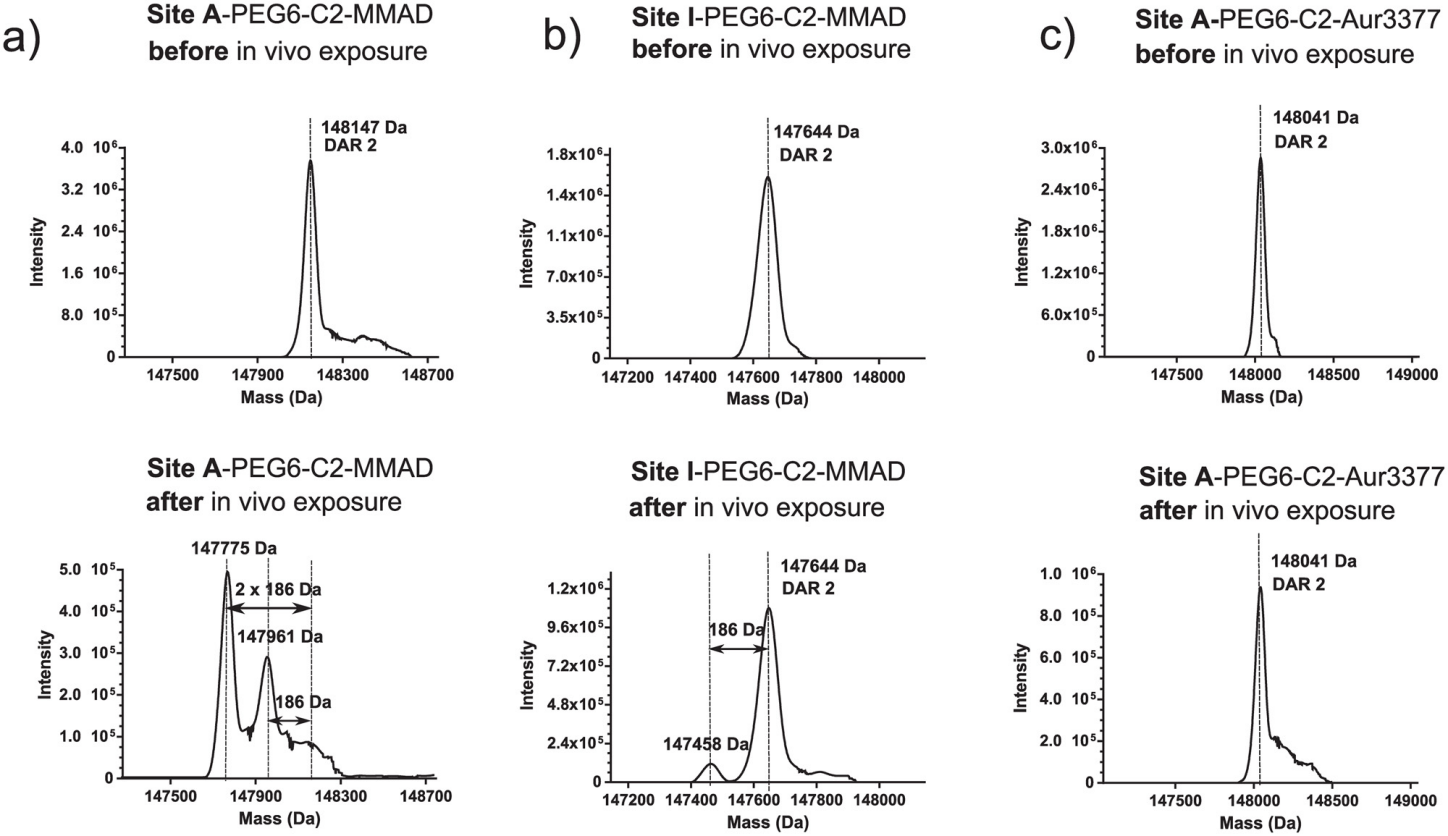
Mass spectrometric analysis of non-cleavable conjugates. The Fig labels represent experimentally observed masses for conjugates before (upper panel) and after (lower panel) *in vivo* exposure. a) Intact mass deconvolution of C16 Site A-PEG6-C2-MMAD conjugate. The metabolic products of the DAR 2 species show a mass loss from either 1 x 186 Da (one payload) or 2 x 186 Da (both payloads). b) Intact mass of C16 Site I-PEG6-C2-MMAD conjugate. The metabolic product shows a 186 Da loss from one of the conjugated payloads. c) Intact mass of C16 Site A-PEG6-C2-Aur3377 conjugate. The *in vivo* exposed conjugate shows no mass shift compared to the untreated compound.

**Fig 3:**
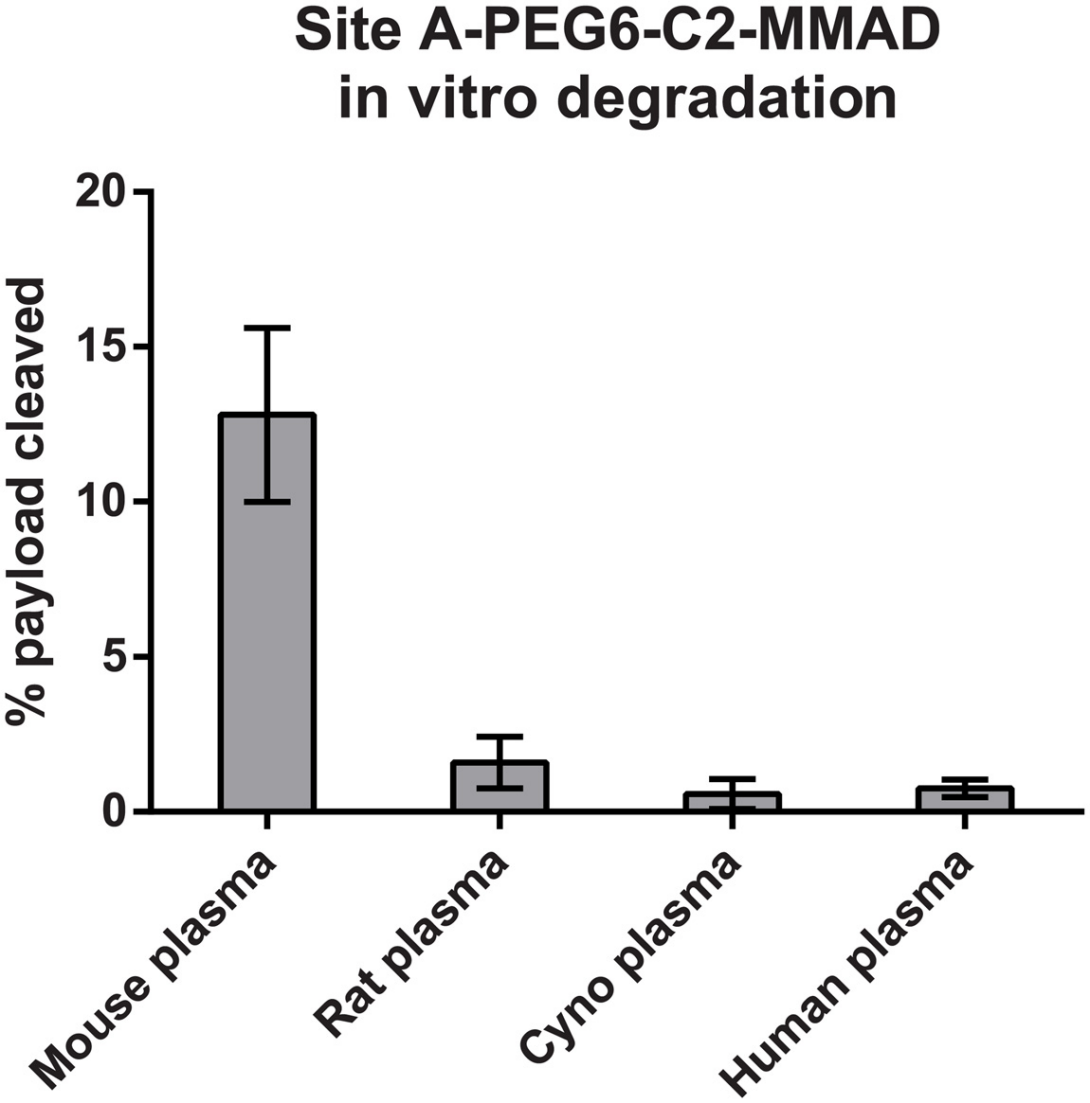
Degradation of the C-terminal portion of the PEG6-C2-MMAD payload in the plasma of different species. Degradation is calculated as percentage of payload cleaved. Calculations are based on DAR values obtained from HIC analysis of the Site A-PEG6-2-MMAD conjugate before and after incubation in plasma for 4.5 days in three independent experiments.

ADC efficacy is determined by the systemic stability of the antibody-drug linkage prior to internalization and its efficient intracellular processing to allow toxin release within the target cell. The high stability of the transglutaminase-generated antibody-drug amide linkage [17, 21] provided us with a unique opportunity to investigate metabolic processes that may be acting upon conjugates with purportedly more stable non-cleavable linker-payloads. Initially, we generated a series of conjugates using the Amino-PEG6-Propionyl-MMAD payload (PEG6-C2-MMAD), a potent analogue of the antimitotic agent Dolastatin 10 [22], attached to specific sites across the anti-M1S1 C16 antibody (Fig 1a and 1b). To evaluate linker-payload stability, we performed comparative *in vitro* plasma stability assays by incubating conjugates in mouse plasma, purifying them and comparing their drug-antibody ratio (DAR) to that of untreated conjugates. Unlike the cleavable Aminocaproyl-valine-citrulline-*p*-aminobenzylcarbamate (C6-VC-PABC) linker [21], the non-cleavable PEG6-C2-MMAD-derived conjugates remained stable across most of the sites tested over a 4.5 day incubation period in mouse plasma, with both the transglutaminase linkage and the Amino-PEG6 linker intact. However, we discovered a position-dependent degradation of the C terminus of MMAD (Fig 1b, Table 1), initially observed by hydrophobic interaction chromatography (HIC) as a decrease in retention time of the ADC following plasma exposure. We subsequently identified the degradation by mass spectrometry as a loss of 186 Da, which is consistent with the hydrolytic cleavage of the C-terminal dolaphenine residue of MMAD (Fig 2a and 2b, Figs a and b in S1 Fig). In contrast to the cleavage observed in mouse plasma, the degradation of the MMAD C terminus was minimal to undetectable in rat, cynomolgus monkey, and human plasma *in vitro* under the conditions studied (Fig 3). Because HIC analysis showed a relatively low, but consistent, degree of MMAD cleavage in the rat plasma, we wanted to verify the identity of the low abundance metabolite. The degraded subpopulation of the C16 Site A-PEG6-C2-MMAD conjugate purified from rat plasma was enriched using HIC, and analyzed by mass spectrometry. MS/MS analysis of tryptic peptide containing the MMAD metabolite revealed the expected fragmentation, confirming that the cleavage in rat plasma is identical to that seen in mouse plasma (Figs b and c in S1 Fig). The conjugates with the lowest and highest stability in mouse plasma, Site A and Site I-PEG6-C2-MMAD, respectively, were then selected for a comparative *in vivo* stability study in the mouse. The loss of the C-terminal residue appears more pronounced *in vivo* in the mouse at the positions tested, as shown by HIC and mass spectrometric analysis of conjugates purified 4.5 days after injection in mice (Table 1, Fig a in S2 Fig).

**Table 1:** Stability of the non-cleavable PEG6-C2-MMAD and PEG6-C2-Aur3377 conjugates under *in vitro* and *in vivo* conditions.

Site	Position	Payload	Intact payload in mouse plasma (%)	Intact payload in mouse in vivo (%)
A	LC 200–202	PEG6-C2-MMAD	87 ± 1.6	20
A	LC 200–202	PEG6-C2-Aur3377	100 ± 0.1	100
B	HC 160	PEG6-C2-MMAD	94 ± 0.3	-
C	HC 135	PEG6-C2-MMAD	96 ± 0.5	-
D	HC C-terminus	PEG6-C2-MMAD	78 ± 1.0	-
D	HC C-terminus	PEG6-C2-Aur3377	100 ± 0.1	-
E	HC 190–192	PEG6-C2-MMAD	91 ± 1.3	-
E	HC 190–192	PEG6-C2-Aur3377	100 ± 0.2	-
F	LC C-terminus	PEG6-C2-MMAD	96 ± 0.4	-
G	N297A	PEG6-C2-MMAD	100 ± 0	-
H	N297Q	PEG6-C2-MMAD	100 ± 0	-
I	HC 294–297	PEG6-C2-MMAD	100 ± 0	93

The percentage of intact MMAD was calculated as the ratio of treated DAR to untreated DAR. Degradation of the C-terminal portion of the PEG6-C2-MMAD payload is considered equivalent to payload removal for DAR determination purposes. Calculations are based on DAR values obtained from HIC analysis for most conjugates, except for Site G, H, and I conjugates for which the percentage of intact MMAD was determined by mass spec analysis. For mouse plasma stability, values reported are averages of three independent experiments. For mouse *in vivo* stability, samples collected from individually dosed mice were pooled to obtain the measurement.

**Fig 4:**
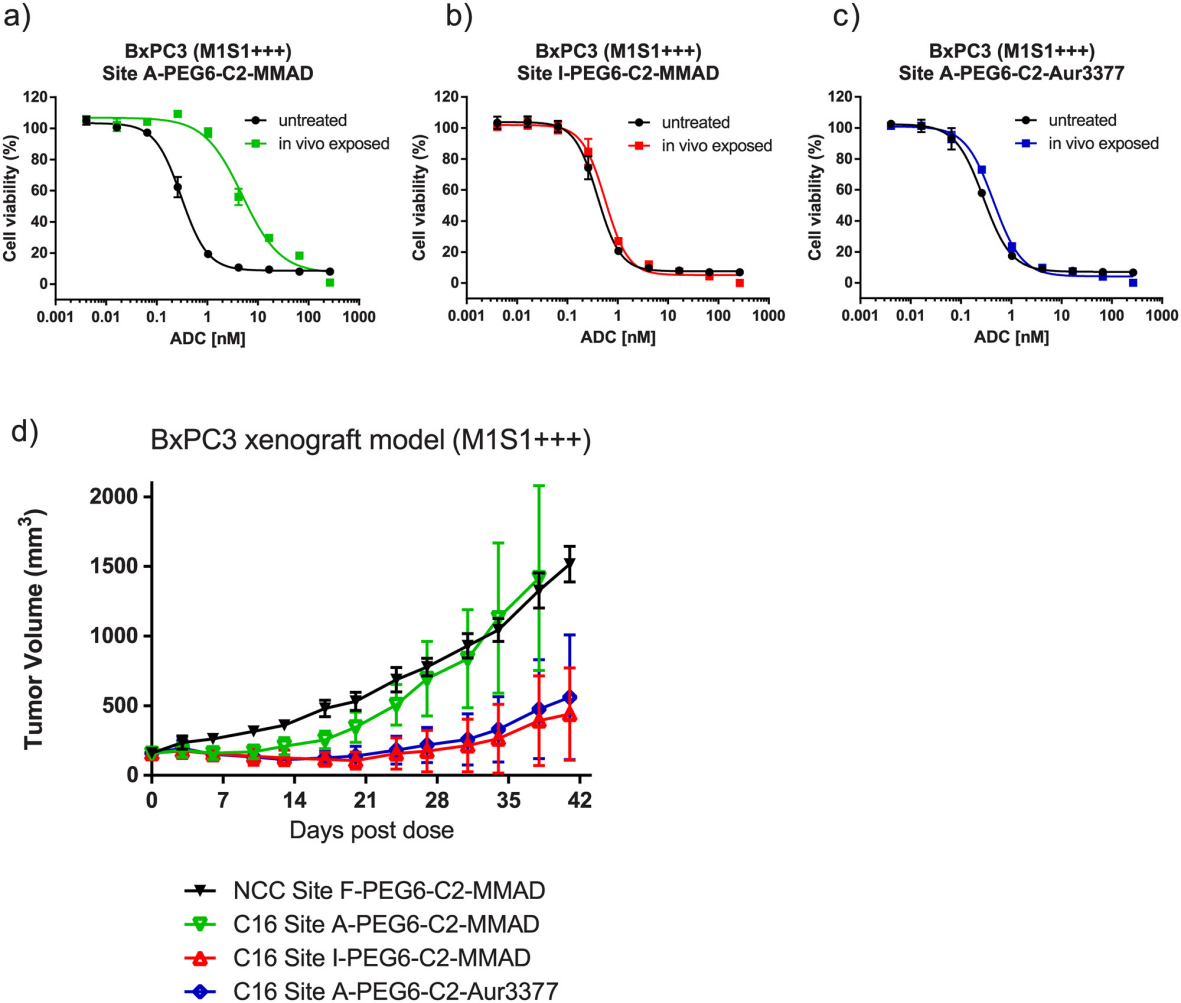
Comparative efficacy studies of non-cleavable ADCs. Comparison of *in vitro* cytotoxic activities of untreated and *in vivo*-exposed non-cleavable conjugates against BxPC3 cells (M1S1+++). a) C16 Site A-PEG6-C2-MMAD conjugate. b) C16 Site I-PEG6-C2-MMAD conjugate. c) C16 Site A-PEG6-C2-Aur3377 conjugate. d) *In vivo* comparison of the three conjugates in the BxPC3 xenograft model, along with a negative control conjugate NCC Site F-PEG6-C2-MMAD. All compounds were given as a single dose at 10 mg/kg.

Since the catabolic process in mouse plasma removes only a small portion of the MMAD payload, we were interested whether the degradation product retains its cytotoxic activity. To assess that, we performed *in vivo* stability experiments by injecting selected PEG6-C2-MMAD conjugates in mice, purifying them from the mouse blood 4.5 days post injection, and comparing their cytotoxicity to the starting material. All of the untreated PEG6-C2-MMAD conjugates showed comparable target-dependent cytotoxicity against the BxPC3 cell line (0.2–0.9 nM) with the highest DAR conjugate showing lowest IC50, and lowest DAR conjugate having highest IC50 (Fig 4a and 4b, S3 Fig, S1 Table). Target specificity was confirmed by lack of efficacy against BxPC3 from a non-binding, negative control conjugate NCC Site F-PEG6-C2-MMAD (Fig a in S3 Fig), and lack of efficacy from all conjugates tested against the non-expressing cell line SW620 (Fig b in S3 Fig). The *in vivo* exposed C16 conjugate Site A-PEG6-C2-MMAD was degraded at the C terminus (Table 1), and showed a significantly reduced *in vitro* cytotoxic activity (0.3 nM vs. 4.9 nM) against the BxPC3 cell line (Fig 3a, S1 Table). Due to the incomplete degradation of the MMAD payload, it is unknown whether the C terminus removal causes partial or complete abolishment of its cytotoxic potency. In contrast to Site A, no significant reduction in cytotoxicity was observed for the stable Site I-PEG6-C2-MMAD conjugate following *in vivo* exposure (0.4 nM vs. 0.6 nM) (Fig 4b, S1 Table).

We further investigated the dependence of C-terminal MMAD cleavage on the structure of the C-terminal residue by comparison with an MMAF analogue, Aur3377 [23]. This analogue contains a negatively charged phenylalanine residue in place of the C-terminal dolaphenine residue on MMAD, and an N-methyl aminoisobutyric acid residue on the N terminus, rather than the N-methyl valine present in MMAD and MMAF (Fig 1c). The conjugates made with Aur3377 retain *in vitro* cytotoxic potency equivalent to MMAD, as demonstrated for Site A-PEG6-C2-MMAD and Site A-PEG6-C2-Aur3377 with identical IC50 of 0.3 nM (Fig 4a and 4c, S1 Table). In contrast to the PEG6-C2-MMAD conjugate, the PEG6-C2-Aur3377 conjugate does not show any detectable degradation in mouse plasma even at the most susceptible Site A and Site D positions on the antibody (Fig 2c, Fig b in S2 Fig, Table 1). Consequently, the cytotoxic potential of Aur3377 is preserved even when incubated for 4.5 days *in vivo* (0.3 nM vs. 0.3 nM, Fig 4c, S1 Table). This suggests that the chemical modifications in the auristatin make the payload incompatible with the binding site of the putative enzyme(s) responsible for the serum degradation of the C terminus of MMAD.

A comparative *in vivo* efficacy study confirmed that the position-dependent C-terminal residue clipping in certain PEG6-C2-MMAD conjugates results in a reduced potency in the BxPC3 mouse xenograft tumor model. Following a single intravenous dose of 10 mg/kg, the ring clipping-sensitive Site A-PEG6-C2-MMAD conjugate showed a strongly reduced *in vivo* efficacy as compared to the stable Site I-PEG6-C2-MMAD or to the degradation-resistant Site A-PEG6-C2-Aur3377 (Fig 4d). As a negative control, we used the NCC Site F-PEG6-C2-MMAD conjugate which is expected to retain most of the intact payload at the relatively stable Site F while in circulation, but it is not expected to bind to target tumor cells (Fig 4d).

To assess whether an enzyme indeed might be involved in the C terminus degradation of PEG6-C2-MMAD in rodent plasma, we carried out a series of inhibition assays using the sensitive Site A conjugate as substrate. Pre-incubation of mouse plasma at 65°C for 1 hour eliminated the C-terminal cleavage of PEG6-C2-MMAD (Table 2), suggesting that a heat-sensitive hydrolase is likely responsible for the degradation process. To classify the putative enzyme, we conducted a series of protease inhibition assays in mouse plasma. Among the protease inhibitors tested, only Pefabloc, mainly a serine protease inhibitor [24], resulted in a complete inhibition of MMAD C-terminal clipping (Table 2). Addition of 3,4-Dichloroisocoumarin, a serine hydrolase inhibitor [25], resulted in partial inhibition of C-terminal MMAD cleavage (Table 2). Other serine protease inhibitors tested (Leupeptin, Antipain, Chymostatin, Aprotinin, and Benzamidine [24, 25]) had no effect on the MMAD degradation (Table 2). Among inhibitors against cysteine-based hydrolases (E64, Leupeptin, Antipain, Chymostatin, and N-ethylmaleimide [24, 25]), only N-ehtylmaleimide resulted in a reduced MMAD C-terminal cleavage (Table 2). Although cysteine-based catalysis cannot be excluded, the plasma enzyme sensitivity to N-ethylmaleimide could be due to reactivity with cysteines, histidines or α-amino group of N-terminal residue [26]. Inhibitors of aspartate proteases (Pepstatin [24]) metalloproteases (Bestatin, EDTA [24]), and carboxypeptidases (carboxypeptidase inhibitor [25]) had no effect on the C-terminal cleavage of MMAD in mouse plasma (Table 2). These data suggest that the MMAD degradation in mouse plasma is most likely mediated by a serine hydrolase mechanism.

**Table 2:** Protease inhibition studies of the PEG6-C2-MMAD degradation in mouse plasma.

Inhibitor	Inhibitor specificity	Payload cleavage in mouse plasma
preheat plasma at 65°C for 1 hour	-	no
E64, 30 uM	Cys proteases & trypsin	yes
Leupeptin, 100 uM	Cys & Ser proteases	yes
Antipain, 100 uM	Cys & Ser proteases	yes
Chymostatin, 100 uM	Cys & Ser proteases	yes
N-ethylmaleimide, 1 mM	Cys proteases	partial
Aprotinin, 5 uM	Ser proteases	yes
Pefabloc, 1 mM	Ser proteases	no
Benzamidine, 4 mM	Ser proteases	yes
3,4-Dichloroisocoumarin, 1 mM	Ser proteases	partial
Pepstatin A, 1 uM	Asp proteases	yes
Bestatin, 135 uM	Metalloproteases	yes
EDTA, 5 mM	Metalloproteases	yes
Carboxypeptidase inhibitor, 10 uM	Carboxypeptidase A & B	yes

“Yes” indicates the same extent of cleavage as observed in plasma without inhibitors, “partial” indicates reduced cleavage compared to uninhibited plasma, while “no” indicates that no degradation was observed. All assays were carried out at pH 7.4.

## Conclusions

Non-cleavable linkers are utilized in roughly 20% of the ADCs presently in the clinic [7], however, the non-cleavable technologies currently undergoing clinical evaluation show various levels of linker instabilities, both pre-clinically as well as in the clinic [27–30]. Several new technologies have been recently developed [12, 14, 15], which aim at improving the stability of non-cleavable linkers. We utilized a transglutaminase-based site-specific conjugation method which generates a stable amide linkage between a primary amine of the linker-payload and a glutamine side chain on the antibody [16, 17]. The created linkage remains inert in plasma environment (both *in vitro* and *in vivo*), which allowed us to systematically examine metabolic processes acting upon the auristatin payload itself across multiple conjugation sites.

We found that the transglutaminase isopeptide linkage and the Amino-PEG6 linker of the non-cleavable PEG6-C2-MMAD conjugates were very stable in mouse, rat, cynomolgus monkey, and human plasma. The high stability of this linkage allowed us to detect degradation of the C-terminal dolaphenine moiety of the MMAD toxin upon exposure to rodent plasma. The extent of MMAD metabolism in the plasma depends on the site of conjugation, with certain sites offering more protection than others. Although it is either absent or greatly reduced in primates *in vitro*, the problem of premature toxin degradation in the circulation appears most severe in mice, and can confound mouse efficacy studies. Understanding these differences among species and eliminating the cleavage mechanism in rodents is essential for preclinical efficacy and safety studies. The cleavage process appears more efficient *in vivo* than *in vitro*, suggesting that the enzyme involved either becomes inactivated *in vitro* and needs to be replenished, or its activity is not optimal under the *in vitro* plasma stability assay conditions, or both. We show that the degradation of the MMAD C terminus, and the resulting loss of cytotoxic potency *in vitro* and *in vivo*, can be prevented by either modifying the toxin which likely interferes with protease processing, or by selecting protective sites for conjugation.

We recently investigated the stability of cleavable linker-payloads at the same positions described here for non-cleavable payloads [21]. Interestingly, there appears to be consistency between the sites that confer the highest stability to the C6-VC-PABC linkage in the cleavable linker-payload and to the C terminus of the PEG6-C2-MMAD linker-payload. This similarity suggests that steric hindrance by the neighboring antibody domains and/or the local environment might be involved in the protective nature of certain sites. The degradation of the MMAD payload may also be carried out by a similar enzymatic class as the plasma protease responsible for the C6-VC-PABC linker cleavage. In fact, both the cleavage of the C6-VC-PABC linker and the degradation of the MMAD C terminus show similar species dependence, and are inhibited by Pefabloc (mainly a serine protease inhibitor) and partially inhibited by 3,4-Dichloroisocoumarin and N-ethylmaleimide, and not affected by other protease inhibitors such as E64, Leupeptin, Antipain, Chymostatin, Aprotinin, Benzamidine, Pepstatin A, Bestatin, EDTA or carboxypeptidase inhibitor (Table 2 and described previously [21]).

The data presented here illustrate the importance of site selection and toxin chemical make up in defining the systemic stability of non-cleavable auristatin-based conjugates. Both the cytotoxic drug and attachment site play crucial role in the stability of conjugated payloads both *in vitro* and *in vivo*, and consequently significantly affect ADC potency. These observations are in agreement with previously published studies describing the dependence of ADC properties on conjugation site [12, 17, 21], and highlight the advantages of the site-specific conjugation approach.

## Materials and Methods

### Protein purification

Anti-M1S1 antibodies were cloned into in-house expression plasmids and transiently expressed in HEK293 cells as previously described [17, 21]. Conditioned media was applied to Protein A MabSelect SuRe columns (GE Healthcare, Inc.) and washed with 140 mM NaCl, 2.7 mM KCl, and 10 mM PO_4_^-3^ (1x PBS) until baseline was reached. Protein was eluted with 100 mM sodium citrate buffer, pH 3.5, and immediately neutralized with 800 mM sodium phosphate buffer, pH 7.4. Eluted protein was dialyzed into 1x PBS and stored at 4°C.

### Conjugation

For the conjugation of anti-M1S1 C16 constructs to Amino-PEG6-C2-MMAD or Amino-PEG6-C2-Aur3377, the antibody concentration was adjusted to 5 mg/mL in buffer containing 25 mM Tris-HCl, pH 8.0 (for PEG6-C2-MMAD conjugations) or pH 8.5 (for PEG6-C2-Aur3377 conjugations), 150 mM sodium chloride, and 100 mM sodium sulfate (for PEG6-C2-Aur3377 conjugations). Payload was added in a 10 to 25-fold molar excess over antibody, and the enzymatic reaction was initiated by addition of 2% (w/v) bacterial transglutaminase (Ajinomoto Activa TI, Japan). Following incubation with shaking at 37°C for 16–24 hours, conjugates were purified using MabSelect SuRe (GE Healthcare, Inc.) following standard procedures. Alternatively, conjugates were purified using preparative Butyl Sepharose™ High Performance (Butyl HP, GE Healthcare Biosciences) by adjusting the reaction mixture to obtain a buffer composition of 0.75 M ammonium sulfate, 25 mM potassium phosphate, pH 7 (Buffer A). The material was applied to a Butyl HP column, washed with 5 column volumes of Buffer A, and eluted with a linear gradient into 25 mM potassium phosphate, pH 7. Fractions containing the ADC were pooled, dialyzed against PBS, concentrated using a 10 kDa Amicon Ultra centrifugal filter unit (Millipore Corporation), and sterile filtered through a 0.2 μm filter.

### Hydrophobic interaction chromatography analysis

Drug-antibody ratios (DAR) for purified conjugates and their metabolic products were evaluated using a TSK-GEL Butyl-NPR column (4.6 mm x 3.5 cm) (Tosoh Bioscience) on an Agilent HP 1100 HPLC (Agilent). The HIC method utilized a mobile phase of 1.5 M ammonium sulfate, 50 mM potassium phosphate, pH 7 for Buffer A, and 50 mM potassium phosphate, pH 7 with 20% isopropanol for Buffer B. Using a flow rate of 0.8 mL/min, ADC in 0.75 M ammonium sulfate was loaded onto the column, and eluted with a gradient consisting of a 2.5 min hold at 0% Buffer B, followed by a 35 minute linear gradient into 100% Buffer B.

### LC/MS intact mass analysis

Prior to LC/MS analysis, conjugates and metabolites were deglycosylated with PNGase F (NEB, cat#P0704L) under non-denaturing conditions at 37°C overnight. ADCs (500 ng) were loaded into a reverse phase column packed with a polymeric material (Michrom-Bruker, cat# CM8/00920/00). LC/MS analysis was performed using Agilent 1100 series HPLC system, comprising binary HPLC pump, degasser, thermostatted auto sampler, column heater and diode-array detector (DAD), coupled to an Orbitrap Velos Pro (Thermo Scientific) mass spectrometer with electrospray ion source. The resulting mass spectra were deconvoluted using ProMass software (Thermo Fisher Scientific).

### LC/MS/MS analysis of ADC tryptic digestions

ADCs (100 μg) were solubilized in 0.2% RapiGest (Waters Corp, 186001861) in 20 mM ammonium bicarbonate. The samples were then incubated at 80°C for 15 min. Dithiothreitol (DTT, 20 mM final concentration) was added and the samples were incubated at 60°C for 30 min to reduce disulfide bonds. After the samples were cooled to room temperature, iodoacetamide (IAA, 15 mM final concentration) was added, and samples were incubated at room temperature for 30 min in the dark to alkylate reduced cysteines. Modified trypsin (Promega, cat#V5111) was added (1:100 enzyme/substrate) and the samples were incubated at 37°C overnight. To hydrolyze the RapiGest prior to mass spectrometry analysis, TFA was added to a final concentration of 60 mM, and the solution was incubated at 37°C for 45 min, and then centrifuged at 20,800 g at 4°C for 30 min. Samples (2.5 μg of digested protein) were loaded into an Agilent Poroshell 120 C18 column (2.1 x 100mm, 2.7μm), and eluted at 45°C with a flow rate of 0.3 mL/min. LC/MS/MS analysis was performed using Agilent 1100 series HPLC system, coupled to an Orbitrap Velos Pro (Thermo Scientific) mass spectrometer with electrospray ion source. Peptides were separated by gradient elution using water as mobile phase A, and acetonitrile as mobile phase B, both containing 0.1% formic acid. The gradient elution used was as follows: 0–2 min 3% B; 2–85 min 3%-50% B; 83–83.1 min 50%-95% B; 83.1–85 min 95% B; 85–85.1 min 95–3% B and 85.1–90 min 3% B. The Orbitrap Velos was operated in information dependent acquisition (IDA) mode with CID performed on the top 10 ions. The MS scan was performed in the Orbitrap at 100,000 resolution, whereas the fragment spectra were collected in the low pressure trap. Ion trap and Orbitrap maximal injection times were set to 50 ms and 500 ms, respectively. The ion target values were 30,000 for the ion trap, and 1,000,000 for the Orbitrap.

### *In vitro* stability and protease inhibitor assays

Plasma samples from mouse, rat, cynomolgus monkey and human were purchased from BioreclamationIVT. *In vitro* stability assays were carried out by incubating 0.125 mg/mL of ADC in 62.5% (v/v) plasma diluted in 1x PBS. Protease inhibitors (Roche, Sigma) were added to plasma at appropriate concentrations as needed. In some assays, pH was adjusted by addition of 120 mM potassium phosphate pH 7.4. Following incubation at 37°C for 3–4.5 days, samples were diluted with an equal volume of 1x PBS, and conjugates were isolated using MabSelect SuRe beads or M1S1 antigen coupled to CNBr-activated Sepharose (GE Healthcare, Inc.) following standard protocols. DAR values were assessed using HIC or mass spectrometric methods. All reported values are averages of three independent measurements.

### *In vivo* stability assays

All animal work in this study was performed in a facility accredited by Association for Assessment and Accreditation of Laboratory Animal Care International (AAALAC International). All protocols were approved by the Rinat Institutional Animal Care and Use Committee (IACUC). All procedures were performed under isoflurane anesthesia, and all efforts were taken to minimize suffering. Our facility complies with the Guide For The Care and Use of Laboratory Animals (Eight Edition). Compounds were dosed IV into 9 weeks old female CB17 SCID mice (The Jackson Laboratory) in 3 groups (n = 9) through the lateral tail vein at 10 mg/kg. Plasma samples were obtained under anesthesia at terminal bleeds 4.5 days following injection. Samples were pooled, diluted with an equal volume of 1x PBS pH 7.4, and conjugates were isolated using MabSelect SuRe beads or M1S1 antigen coupled to CNBr-activated Sepharose (GE Healthcare, Inc.) following standard protocols. DAR values were assessed using HIC or mass spectrometric methods.

### *In vitro* cytotoxicity assays

*In vitro* cytotoxicity studies of anti-M1S1 ADCs were performed on the high target-expressing pancreatic adenocarcinoma BxPC3 cell line (M1S1+++), and the target-negative colorectal adenocarcinoma SW620 cell line (M1S1-). Cells were seeded on white walled clear bottom plates at 2000 cells per well 24 hours before treatment. Cells were treated with 4-fold serially diluted ADCs in triplicates. Cell viability was determined by CellTiter-Glo Luminescent Cell Viability Assay 96 (Promega, Madison, WI) 96 hours after treatment. Relative cell viability was determined as percentage of untreated control. IC50 values were calculated by GraphPad Prism 5 software.

### *In vivo* efficacy studies

*In vivo* efficacy studies of ADCs with different stabilities were performed with the high target-expressing BxPC3 (M1S1 +++) xenograft model. Two million BxPC3 cancer cells were implanted subcutaneously under isoflurane anesthesia into 9 weeks old CB17 SCID mice (The Jackson Laboratory) in 3 experimental groups and 1 control group, and tumor growth was monitored until the tumor sizes reached around 200 mm^3^. Animals were randomized by tumor sizes into groups of 4–5, and a single dose of 10 mg/kg of non-cleavable ADC was administered through bolus tail vein injection. Tumor volume was measured twice a week by a Caliper device and calculated with the following formula: Tumor volume = (length x width2) / 2. Animals were euthanized once their tumor volumes reached 2000 mm^3^.

### Chemical synthesis

**Fig 5:**
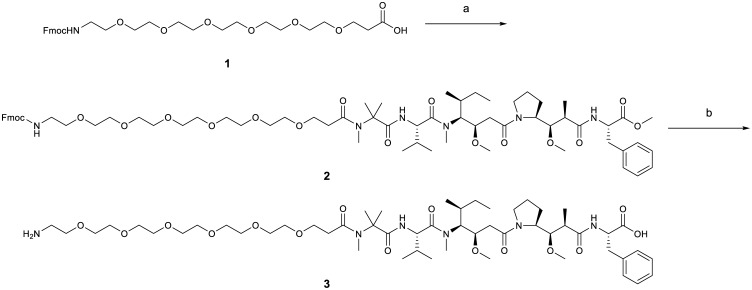
Synthesis of Amino-PEG6-C2-Aur3377. Reagents and conditions: a) Aur-3377, Fmoc-AmPeg6C2-CO2H, HATU, *i*-Pr_2_NEt, DMF; b) LiOH, H_2_O-DMF.

Synthesis of Amino-PEG6-C2-MMAD (AmPeg6C2-MMAD) was previously described [17]. Synthesis of Amino-PEG6-C2-Aur3377 (AmPeg6C2-Auristatin-3377, **3**) was as follows (Fig 5):

#### *N*-[1-(*9H*-fluoren-9-yl)-3,25-dioxo-2,7,10,13,16,19,22-heptaoxa-4-azapentacosan-25-yl]-*N*,2-dimethylalanyl-N-[(*3R*, *4S*, *5S*)-3-methoxy-1-{(*2S*)-2-[(*1R*, *2R*)-1-methoxy-3-{[(*2S*)-1-methoxy-1-oxo-3-phenylpropan-2-yl]amino}-2-methyl-3-oxopropyl]pyrrolidin-1-yl}-5-methyl-1-oxoheptan-4-yl]-*N*-methyl-L-valinamide (Fmoc-AmPeg6C2-Aur-3377, 2)

To a solution of *N*,2-dimethylalanyl-*N*-[(*3R*, *4S*, *5S*)-3-methoxy-1-{(*2S*)-2-[(*1R*, *2R*)-1-methoxy-3-{[(*2S*)-1-methoxy-1-oxo-3-phenylpropan-2-yl]amino}-2-methyl-3-oxopropyl]pyrrolidin-1-yl}-5-methyl-1-oxoheptan-4-yl]-N-methyl-L-valinamide [23] (560 mg, 0.765 mmol) in *N*, *N*-dimethylformamide (4 mL) were added Fmoc-21-amino-4,7,10,13,16,19-hexaoxaheneicosanoic acid (Fmoc-AmPeg6C2CO2H, 661 mg, 1.15 mmol), HATU (445 mg, 1.15 mmol), and then *N*, *N*-diisopropylethylamine (0.405 mL, 200 mg, 2.3 mmol) and the mixture was allowed to stir overnight at room temperature. Solvent was then removed *in vacuo* and the resulting residue was purified by HPLC over a 100 x 30 mm, 10 μm Phenomenex Luna C18 column using flow rate of 20 mL/minute and gradient elution of 30–95% acetonitrile in water containing 0.02% acetic acid over 20 minutes. Pool product containing fractions were lyophilized to provide 600 mg (61%) of the desired product (**7**) as white solid. MS (ES): *m/z* 1289.8 [M+H]^+^.

#### *N*-(21-amino-4,7,10,13,16,19-hexaoxahenicosan-1-oyl)-*N*,2-dimethylalanyl-*N*-[(*3R*, *4S*, *5S*)-1-{(*2S*)-2-[(*1R*, *2R*)-3-{[(*1S*)-1-carboxy-2-phenylethyl]amino}-1-methoxy-2-methyl-3-oxopropyl]pyrrolidin-1-yl}-3-methoxy-5-methyl-1-oxoheptan-4-yl]-*N*-methyl-L-valinamide (AmPeg6C2-Auristatin-3377, 3)

To a solution of *N*-[1-(*9H*-fluoren-9-yl)-3,25-dioxo-2,7,10,13,16,19,22-heptaoxa-4-azapentacosan-25-yl]-*N*,2-dimethylalanyl-N-[(*3R*, *4S*, *5S*)-3-methoxy-1-{(*2S*)-2-[(*1R*, *2R*)-1-methoxy-3-{[(*2S*)-1-methoxy-1-oxo-3-phenylpropan-2-yl]amino}-2-methyl-3-oxopropyl]pyrrolidin-1-yl}-5-methyl-1-oxoheptan-4-yl]-*N*-methyl-L-valinamide (**2**, 705 mg, 0.546 mmol) in *N*, *N*-dimethylformamide (8 mL) was added a solution of LiOH (162 mg, 6.76 mmol) in water (2 mL), and the mixture was stirred at room temperature for 20 minutes. The pH was then adjusted pH ~5 by addition of AcOH (pH paper) and the entire reaction mixture was then purified directly by reverse phase chromatography over a 100 x 30 mm, 5 μm Phenomenex Luna C18 column using flow rate of 20 mL/minute and gradient elution of 10–75% acetonitrile in water containing 0.02% acetic acid over 20 minutes. Pooled product-containing fractions were concentrated and lyophilized to provide 389 mg (64%) of the desired product (**3**) as a white solid. MS (ES): *m/z* 1053.8 [M+H]^+^. This material was greater than 95% pure by analytical HPLC and the ^1^H NMR was consistent with the assigned structure.

## Supporting Information

S1 Checklist The ARRIVE Guidelines Checklist—Animal Research. (PDF)

S1 Fig Mass spectrometric analysis of the metabolic product of C16 Site A-PEG6-C2-MMAD conjugate following *in vitro* exposure. (JPG)

S2 Fig HIC characterization of *in vivo* stabilities of non-cleavable conjugates. (JPG)

S3 Fig Cytotoxicity assays of non-cleavable PEG6-C2-MMAD conjugates across various sites. (JPG)

S1 Table Cytotoxicity against the BxPC3 cells (M1S1+++) of non-cleavable conjugates before and after *in vivo* exposure. (JPG)
