# Supplementary material for: Site-Dependent Degradation of a Non-Cleavable Auristatin-Based Linker-Payload in Rodent Plasma and Its Effect on ADC Efficacy
Source: PLoS One. 2015 Jul 10;10(7):e0132282. doi: 10.1371/journal.pone.0132282 (PMC4498778; doi:10.1371/journal.pone.0132282)
Supplement: S1 Checklist — (PDF) [file pone.0132282.s001.pdf]

The ARRIVE Guidelines Checklist

Animal Research: Reporting In Vivo Experiments

Carol Kilkenny[1], William J Browne[2], Innes C Cuthill[3], Michael Emerson[4] and Douglas G Altman[5]

[1]The National Centre for the Replacement, Refinement and Reduction of Animals in Research, London, UK, [2]School of Veterinary Science, University of Bristol, Bristol, UK, [3]School of Biological Sciences, University of Bristol, Bristol, UK, [4]National Heart and Lung Institute, Imperial College London, UK, [5]Centre for Statistics in Medicine, University of Oxford, Oxford, UK.

	ITEM	RECOMMENDATION	Section/ Paragraph
Title	1	Provide as accurate and concise a description of the content of the article as possible.	Title page
Abstract	2	Provide an accurate summary of the background, research objectives, including details of the species or strain of animal used, key methods, principal findings and conclusions of the study.	Abstract
**INTRODUCTION**			
Background	3	a. Include sufficient scientific background (including relevant references to previous work) to understand the motivation and context for the study, and explain the experimental approach and rationale.<br>b. Explain how and why the animal species and model being used can address the scientific objectives and, where appropriate, the study's relevance to human biology.	Introduction
Objectives	4	Clearly describe the primary and any secondary objectives of the study, or specific hypotheses being tested.	See attached
**METHODS**			
Ethical statement	5	Indicate the nature of the ethical review permissions, relevant licences (e.g. Animal [Scientific Procedures] Act 1986), and national or institutional guidelines for the care and use of animals, that cover the research.	Methods
Study design	6	For each experiment, give brief details of the study design including:<br>a. The number of experimental and control groups.<br>b. Any steps taken to minimise the effects of subjective bias when allocating animals to treatment (e.g. randomisation procedure) and when assessing results (e.g. if done, describe who was blinded and when).<br>c. The experimental unit (e.g. a single animal, group or cage of animals).<br>A time-line diagram or flow chart can be useful to illustrate how complex study designs were carried out.	Results<br>Methods
Experimental procedures	7	For each experiment and each experimental group, including controls, provide precise details of all procedures carried out. For example:<br>a. How (e.g. drug formulation and dose, site and route of administration, anaesthesia and analgesia used [including monitoring], surgical procedure, method of euthanasia). Provide details of any specialist equipment used, including supplier(s).<br>b. When (e.g. time of day).<br>c. Where (e.g. home cage, laboratory, water maze).<br>d. Why (e.g. rationale for choice of specific anaesthetic, route of administration, drug dose used).	Methods<br>See attached
Experimental animals	8	a. Provide details of the animals used, including species, strain, sex, developmental stage (e.g. mean or median age plus age range) and weight (e.g. mean or median weight plus weight range).<br>b. Provide further relevant information such as the source of animals, international strain nomenclature, genetic modification status (e.g. knock-out or transgenic), genotype, health/immune status, drug or test naïve, previous procedures, etc.	Methods<br>See attached

Housing and husbandry	9	Provide details of:<br>a. Housing (type of facility e.g. specific pathogen free [SPF]; type of cage or housing; bedding material; number of cage companions; tank shape and material etc. for fish).<br>b. Husbandry conditions (e.g. breeding programme, light/dark cycle, temperature, quality of water etc for fish, type of food, access to food and water, environmental enrichment).<br>c. Welfare-related assessments and interventions that were carried out prior to, during, or after the experiment.	See attached
Sample size	10	a. Specify the total number of animals used in each experiment, and the number of animals in each experimental group.<br>b. Explain how the number of animals was arrived at. Provide details of any sample size calculation used.<br>c. Indicate the number of independent replications of each experiment, if relevant.	See attached
Allocating animals to experimental groups	11	a. Give full details of how animals were allocated to experimental groups, including randomisation or matching if done.<br>b. Describe the order in which the animals in the different experimental groups were treated and assessed.	See attached
Experimental outcomes	12	Clearly define the primary and secondary experimental outcomes assessed (e.g. cell death, molecular markers, behavioural changes).	Results
Statistical methods	13	a. Provide details of the statistical methods used for each analysis.<br>b. Specify the unit of analysis for each dataset (e.g. single animal, group of animals, single neuron).<br>c. Describe any methods used to assess whether the data met the assumptions of the statistical approach.	See attached
RESULTS			
Baseline data	14	For each experimental group, report relevant characteristics and health status of animals (e.g. weight, microbiological status, and drug or test naïve) prior to treatment or testing. (This information can often be tabulated).	See attached
Numbers analysed	15	a. Report the number of animals in each group included in each analysis. Report absolute numbers (e.g. 10/20, not 50%[2]).<br>b. If any animals or data were not included in the analysis, explain why.	See attached
Outcomes and estimation	16	Report the results for each analysis carried out, with a measure of precision (e.g. standard error or confidence interval).	Table 1<br>Figure 4d
Adverse events	17	a. Give details of all important adverse events in each experimental group.<br>b. Describe any modifications to the experimental protocols made to reduce adverse events.	See attached
DISCUSSION			
Interpretation/ scientific implications	18	a. Interpret the results, taking into account the study objectives and hypotheses, current theory and other relevant studies in the literature.<br>b. Comment on the study limitations including any potential sources of bias, any limitations of the animal model, and the imprecision associated with the results[2].<br>c. Describe any implications of your experimental methods or findings for the replacement, refinement or reduction (the 3Rs) of the use of animals in research.	Results<br>Conclusions<br>See attached
Generalisability/ translation	19	Comment on whether, and how, the findings of this study are likely to translate to other species or systems, including any relevance to human biology.	Conclusions & attached
Funding	20	List all funding sources (including grant number) and the role of the funder(s) in the study.	See attached

References:
1. Kilkenny C, Browne WJ, Cuthill IC, Emerson M, Altman DG (2010) Improving Bioscience Research Reporting: The ARRIVE Guidelines for Reporting Animal Research. *PLoS Biol* 8(6): e1000412. doi:10.1371/journal.pbio.1000412
2. Schulz KF, Altman DG, Moher D, the CONSORT Group (2010) CONSORT 2010 Statement: updated guidelines for reporting parallel group randomised trials. *BMJ* 340:c332.

ARRIVE Guidelines Checklist

ITEM	DESCRIPTION
1	Site-dependent degradation of a non-cleavable auristatin-based linker-payload in rodent plasma and its effect on ADC efficacy
2	see Abstract
3a & b	see Introduction
4	Preclinical studies for development of cancer therapeutics utilize rodent species to test novel compounds for efficacy and safety. In order to achieve a cytotoxic effect in the target cells, cancer drugs must be stable in systemic circulation. We examined non-cleavable auristatin-based antibody-drug conjugates (ADCs) for stability in the mouse plasma and in vivo, and demonstrated that changing the position of the drug attachment or the chemical identity of the drug can affect both ADC stability and efficacy in this model organism. The observations have direct implications to preclinical and clinical cancer programs utilizing similar ADC compounds.
5	All animal work in this study was performed in a facility accredited by Association for Assessment and Accreditation of Laboratory Animal Care International (AAALAC International). All protocols were approved by the Rinat Institutional Animal Care and Use Committee (IACUC). All procedures were performed under isoflurane anesthesia, and all efforts were taken to minimize suffering. Our facility complies with the Guide For The Care and Use of Laboratory Animals (Eight Edition).
6	In vivo stability study comprised 3 experimental groups with one ADC compound tested per group. Each group included 9 mice, and collected plasma samples were pooled for averaging purposes prior to further analysis.<br><br>In vivo efficacy study comprised 3 experimental groups and 1 control group. Mice were randomized by tumor sizes into groups of 4-5.
7	Drug formulation, dose, site and route of administration followed standard efficacy study protocols, and were the same for in vivo stability study. Animals were handled in the Rinat Vivarium throughout the studies.
8	In vivo stability study: CB17 SCID mice, female, 9 weeks (same age), mean weight 19.8 g, weight range 18.1-22.3 g, all mice appeared healthy throughout the study.<br><br>In vivo efficacy study: CB17 SCID mice, female, 9 weeks (same age), median weight 22.7 g, weight range 19.3-27.2 g, all mice appeared healthy throughout the study.<br><br>Mice were obtained from the Jackson Laboratory, strain name: CBySmn.CB17-*Prkdc*^{scid}/J. This is the strain of choice for efficacy testing of therapeutic antibodies, according to the vendor. Mice had not had any previous procedures.
9	All experiments took place in a specific pathogen free facility. Mice were housed inside autoclaved Allentown Hepa-filtered ventilated cages. We used irradiated Purina pico lab feed, and Anderson bed o cob 1/8 cut bedding. Four to five mice were housed per cage. Temperature and humidity were monitored and maintained at 68-70 F and 30-70%. No interventions were required prior to, during, or after the experiment.
10	In vivo stability study: 9 animals per group, 27 animals total.<br><br>In vivo efficacy study: 5 animals per group, 20 animals total (selected from a larger cohort).

For in vivo stability study, sample sizes were estimated based on the amount of compound required for cytotoxicity assays and metabolite analysis following plasma collection. For in vivo efficacy study, sample sizes strictly followed an IACUC approved protocol.

The in vivo efficacy study was repeated once to verify the results.

11 For in vivo stability study, no randomization was necessary prior to treatment, and final results were averaged by pooling samples collected from each group.

For in vivo efficacy study, randomization was carried out to achieve the closest average tumor size among groups.

No particular order was required to treat or asses the animals.

12 Primary experimental outcome assessed: stability of ADC compounds isolated from collected mouse plasma, as determined using biochemical methods.

Secondary experimental outcome assessed: efficacy of ADC compounds in mice, as determined by monitoring tumor sizes following a single ADC dose.

13 In the stability study, the biochemical assays used did not rely on statistical analysis.

In the efficacy study, individual measurements were tabulated and plotted using GraphPad Prism software with the provided statistical package.

14 In vivo stability study: all experimental groups appeared healthy before and throughout the study, weight range 18.1-22.3 g, no prior treatments.

In vivo efficacy study: all experimental and control groups appeared healthy before and throughout the study, weight range 19.3-27.2 g, no prior treatments.

15 All animals were included in all analyses in both studies.

16 For in vivo stability study, values reported in Table 1 were obtained from samples averaged by pooling. No standard error is reported.

For in vivo efficacy study, error bars are displayed in Figure 4d.

17 No adverse events were reported in any study.

18 See Results and Discussion, and Conclusions for the interpretation and detailed discussion of in vivo stability and efficacy results. The limitation of the mouse tumor models for development of cancer therapeutics is related to the differences in ADC stability observed among species. We show that the stability of ADC compounds can be improved in the rodents in order to select most potent therapeutics for clinical studies.

We used the minimum number of animals to test the hypothesis of the study.

19 The findings in this study, as pertaining to efficacy and safety of ADC therapeutics in preclinical evaluation, are relevant to any clinical cancer programs utilizing similar compounds.

20 The authors received no specific funding for this work.
