# Supplementary figures and images for: Site-Dependent Degradation of a Non-Cleavable Auristatin-Based Linker-Payload in Rodent Plasma and Its Effect on ADC Efficacy
Source: PLoS One. 2015 Jul 10;10(7):e0132282. doi: 10.1371/journal.pone.0132282 (PMC4498778; doi:10.1371/journal.pone.0132282)

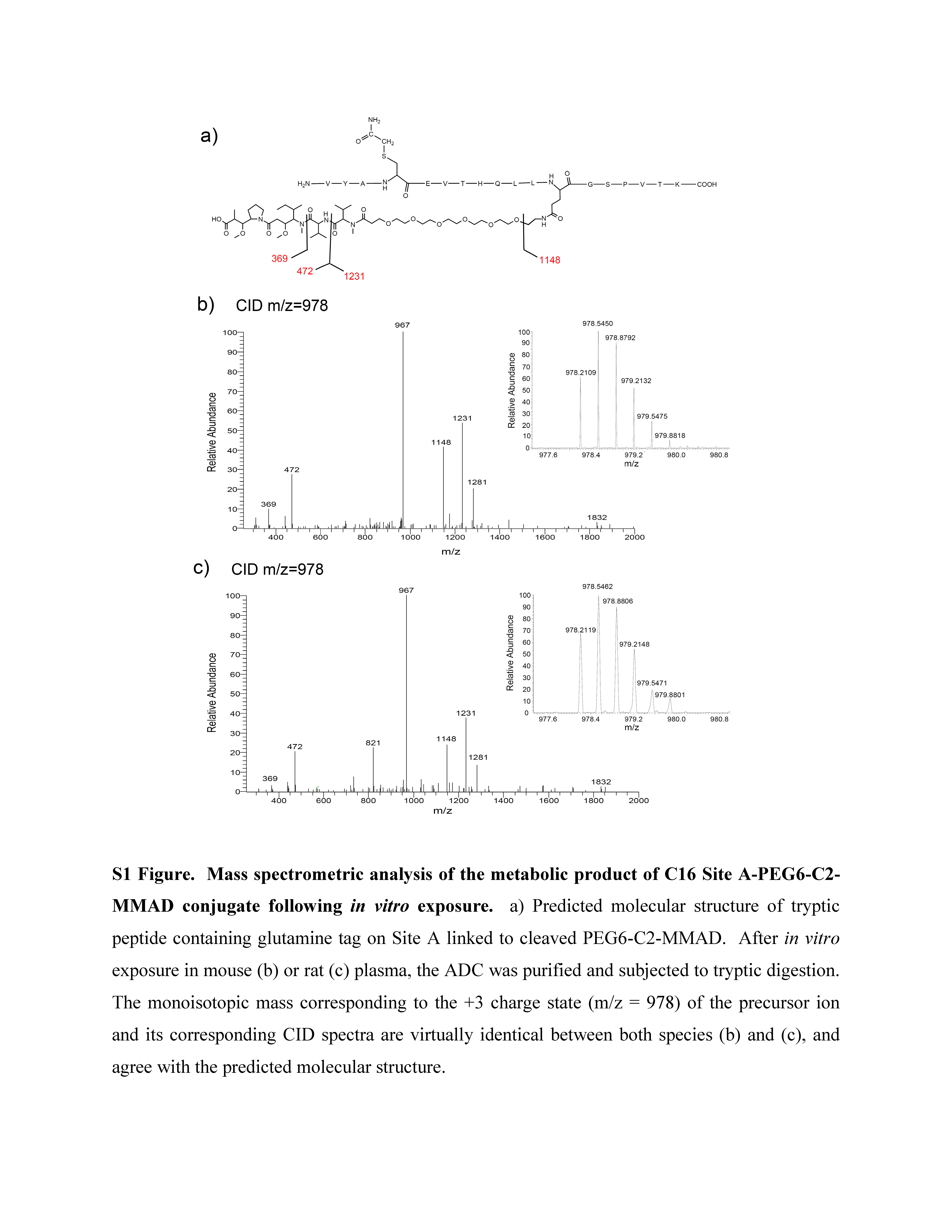

Supplement: S1 Fig — (JPG) [file pone.0132282.s002.jpg]

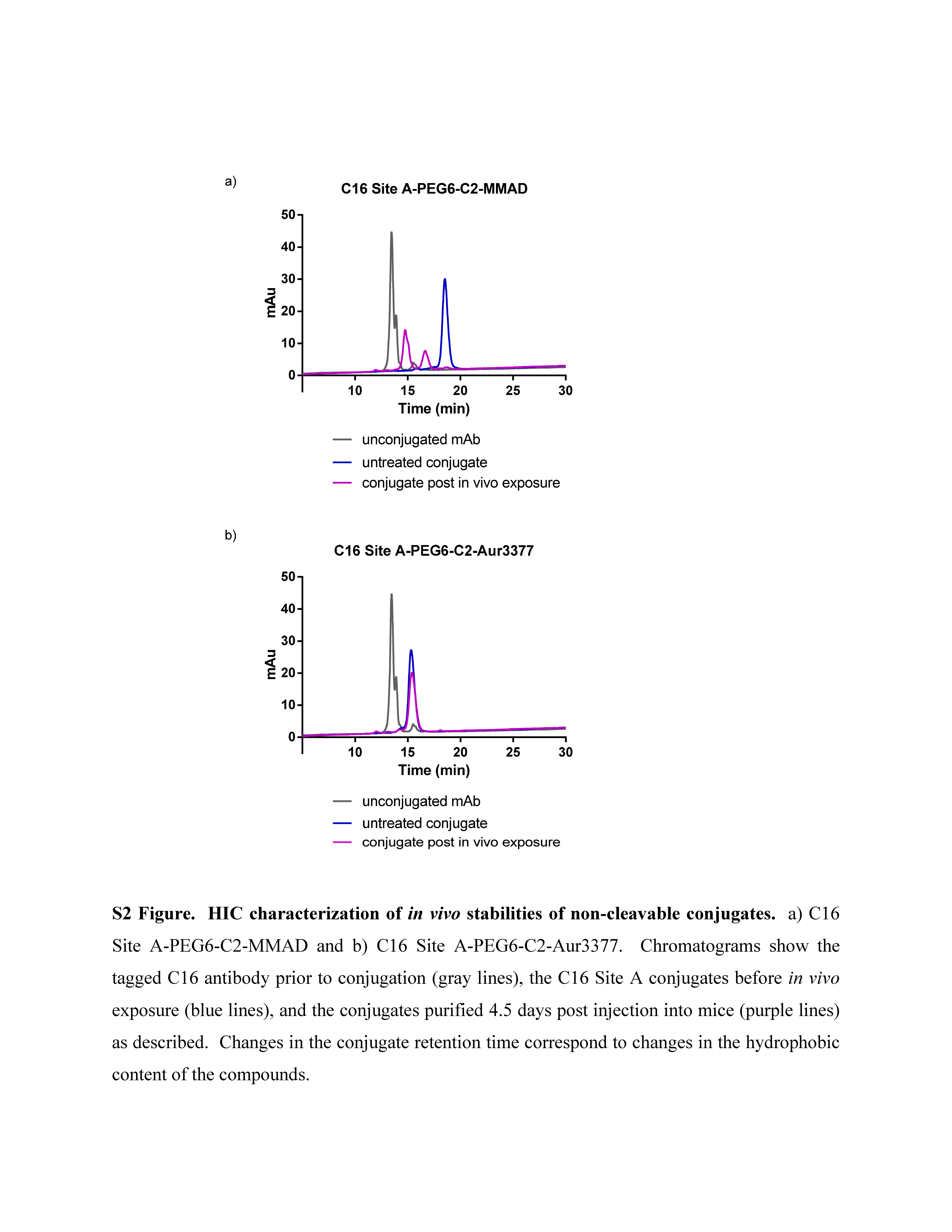

Supplement: S2 Fig — (JPG) [file pone.0132282.s003.jpg]

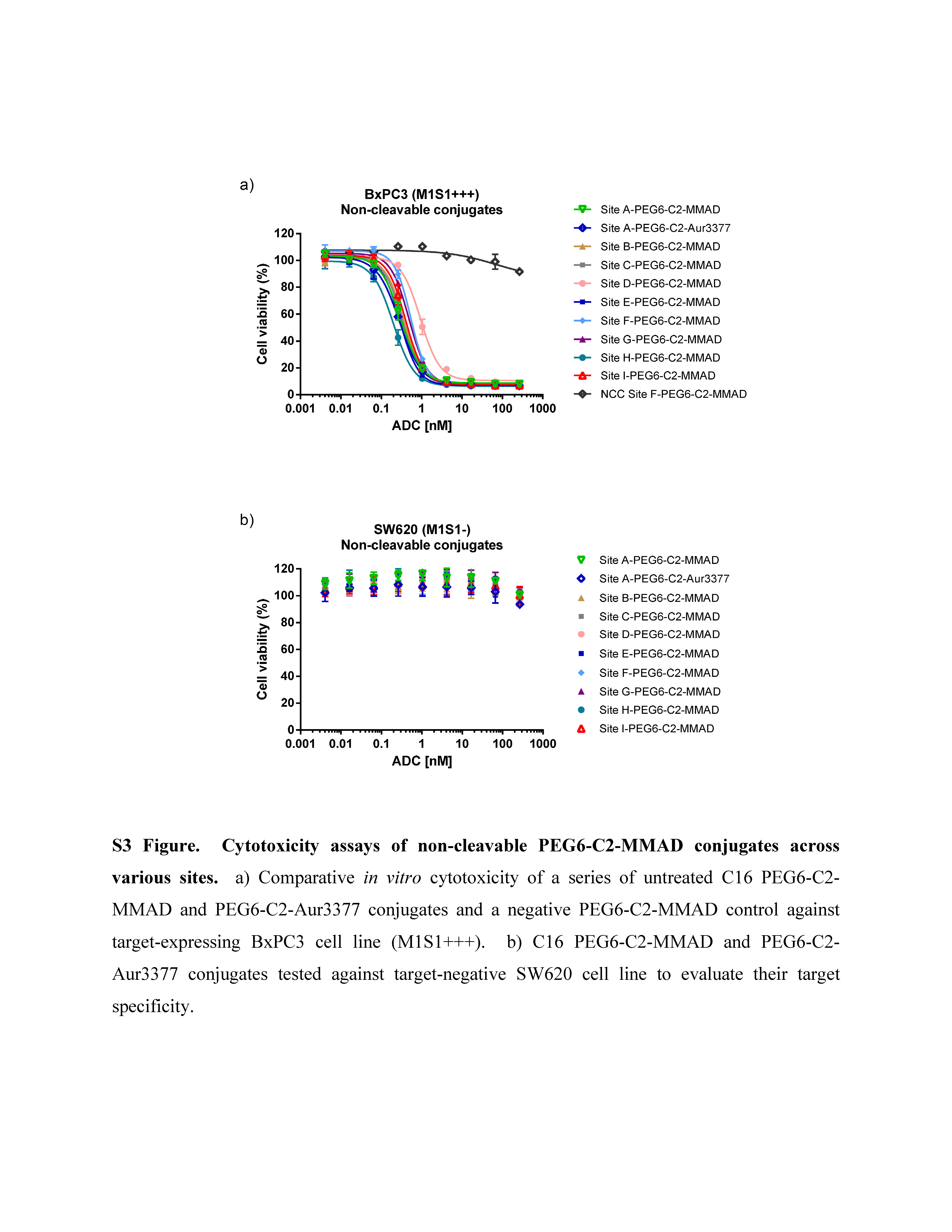

Supplement: S3 Fig — (JPG) [file pone.0132282.s004.jpg]

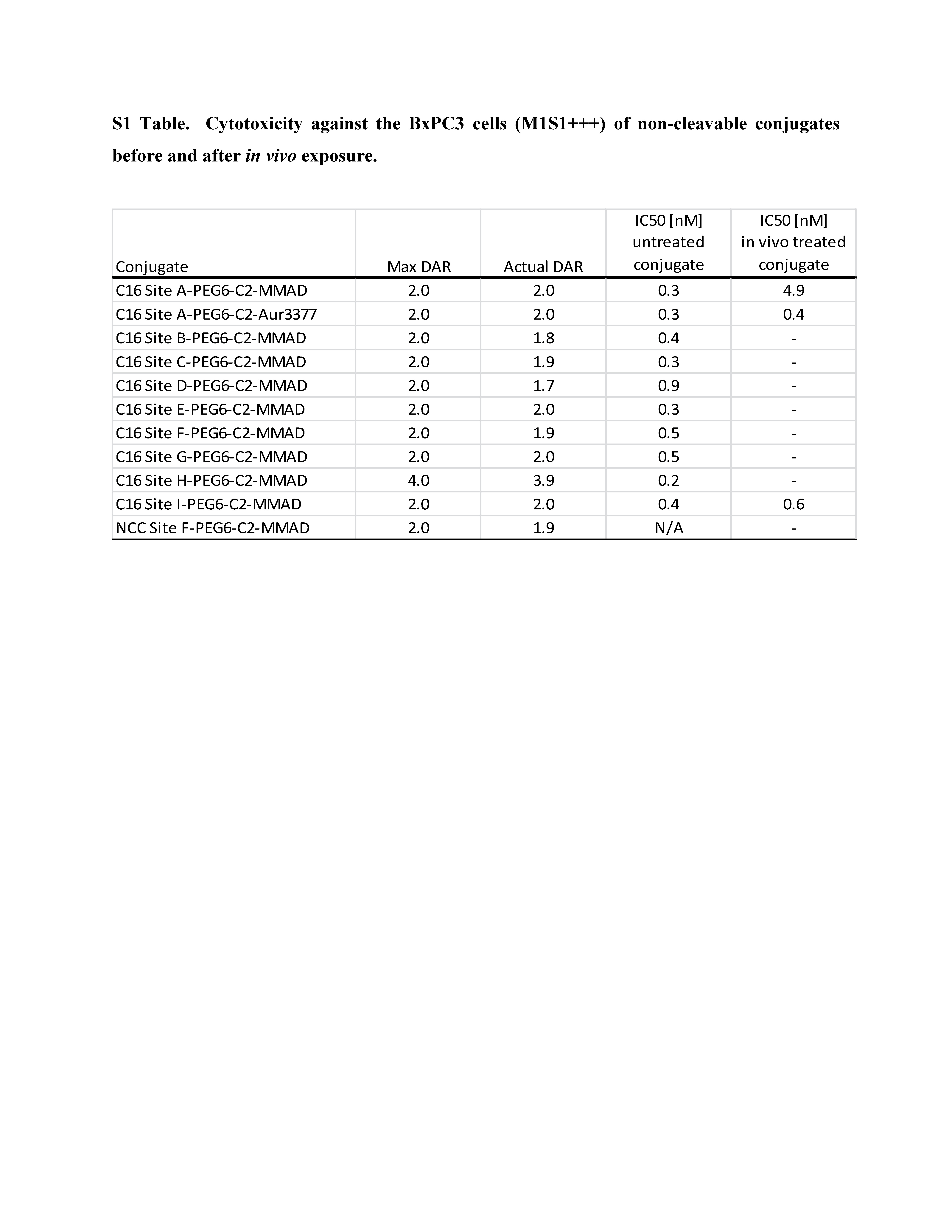

Supplement: S1 Table — (JPG) [file pone.0132282.s005.jpg]
